# [1-Meth­oxy-3-(pyridin-2-yl)indolizin-2-yl](pyridin-2-yl)methanone

**DOI:** 10.1107/S160053681203396X

**Published:** 2012-08-04

**Authors:** Tobias Kloubert, Robert Kretschmer, Helmar Görls, Matthias Westerhausen

**Affiliations:** a Institut für Anorganische und Analytische Chemie, Friedrich-Schiller-Universität Jena, Humboldt-Strasse 8, 07743 Jena, Germany

## Abstract

Methyl­ation of [1-hy­droxy-3-(pyridin-2-yl)indolizin-2-yl](pyridin-2-yl)methanone was performed *via* metalation with potassium *tert*-butano­late in toluene and a subsequent metathesis reaction with methyl iodide yielded the yellow title compound, C_20_H_15_N_3_O_2_. The substituents at the indolizine unit are twisted [the indolizine ring system makes dihedral angles of 34.67 (7) and 77.49 (5)°, respectively, with the pyridyl and pyridinoyl rings] with single bonds between the central unit and the attached pyridine ring [1.459 (3) Å] and the pyridinoyl group [1.483 (3) Å]. There are no classical hydrogen bonds in the crystal structure.

## Related literature

Indolizines are used as dyes (Weidner *et al.*, 1989 ▶), pharmaceuticals (Singh & Mmatli, 2011 ▶), and spectroscopic sensitizers (Gilchrist, 2001 ▶; Katrizky *et al.*, 1999 ▶; Sarkunam & Nallu, 2005 ▶; Vemula *et al.*, 2011 ▶; Weeler, 1985*a* ▶,*b* ▶). Indolizines are rather scarce in nature whereas the reduced form of these heteroaromatic bicyclic compounds, the indolizidines, are quite common, see: Michael (2007 ▶) and references therein. Well defined substitution patterns are required (Sarkunam & Nallu, 2005 ▶; Swinbourne *et al.*, 1978 ▶; Uchida & Matsumoto, 1976 ▶) and therefore, different transition-metal mediated and metal-free strategies for the synthesis of substituted indolizines have been developed (Jacobs *et al.*, 2011 ▶; Swinbourne *et al.*, 1978 ▶; Kel’in *et al.*, 2001 ▶; Kim *et al.*, 2010 ▶; Liu *et al.*, 2007 ▶; Morra *et al.*, 2006 ▶; Seregin & Gevorgyan, 2006 ▶; Yan & Liu, 2007 ▶). Pyridinium *N*-methyl­ides react with acetyl­enes or with ethyl­enes in the presence of an oxidant to make indolizines (Miki *et al.*, 1984 ▶; Padwa *et al.*, 1993; ▶ Wei *et al.*, 1993 ▶). For cyclization of 1,1-diacetyl-2-(2-pyrid­yl)ethyl­ene in acetic acid anhydride or in dimethyl­sulfoxide-yielding indolizines, see: Pohjala (1974 ▶, 1977 ▶).
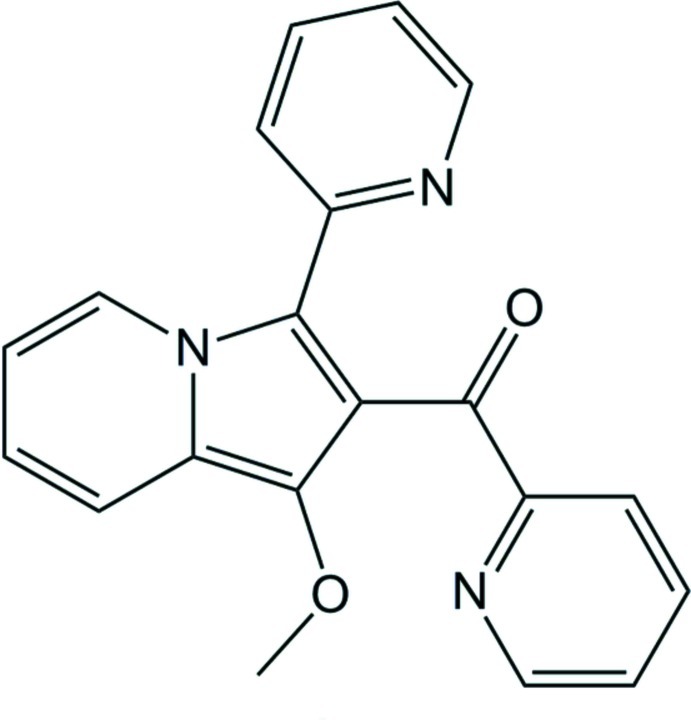


## Experimental

### 

#### Crystal data

C_20_H_15_N_3_O_2_
*M* *_r_* = 329.35
Monoclinic, 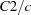

*a* = 25.822 (2) Å
*b* = 11.4406 (9) Å
*c* = 11.3602 (7) Å
β = 107.070 (4)°
*V* = 3208.2 (4) Å^3^
*Z* = 8
Mo *K*α radiation
μ = 0.09 mm^−1^
*T* = 183 K
0.05 × 0.05 × 0.05 mm

#### Data collection

Nonius KappaCCD diffractometer
10598 measured reflections
3667 independent reflections
2207 reflections with *I* > 2σ(*I*)
*R*_int_ = 0.070

#### Refinement

*R*[*F* ^2^ > 2σ(*F* ^2^)] = 0.054
*wR*(*F* ^2^) = 0.132
*S* = 1.03
3667 reflections
275 parameters
H atoms treated by a mixture of independent and constrained refinement
Δρ_max_ = 0.22 e Å^−3^
Δρ_min_ = −0.28 e Å^−3^

### 

Data collection: *COLLECT* (Nonius, 1998 ▶); cell refinement: *DENZO* (Otwinowski, Minor, 1997 ▶); data reduction: *DENZO*; program(s) used to solve structure: *SHELXS97* (Sheldrick, 2008 ▶); program(s) used to refine structure: *SHELXL97* (Sheldrick, 2008 ▶); molecular graphics: *SHELXTL/PC* (Sheldrick, 2008 ▶); software used to prepare material for publication: *SHELXL97*.

## Supplementary Material

Crystal structure: contains datablock(s) I, global. DOI: 10.1107/S160053681203396X/gg2090sup1.cif

Structure factors: contains datablock(s) I. DOI: 10.1107/S160053681203396X/gg2090Isup2.hkl

Supplementary material file. DOI: 10.1107/S160053681203396X/gg2090Isup3.cml

Additional supplementary materials:  crystallographic information; 3D view; checkCIF report

## Figures and Tables

**Fig. 1.**
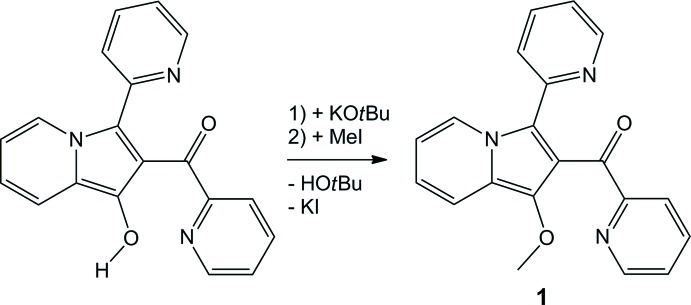
The preparative pathway to Ivia metalation with potassium tert-butanolate and a subsequent metathesis reaction with methyl iodide.

**Fig. 2.**
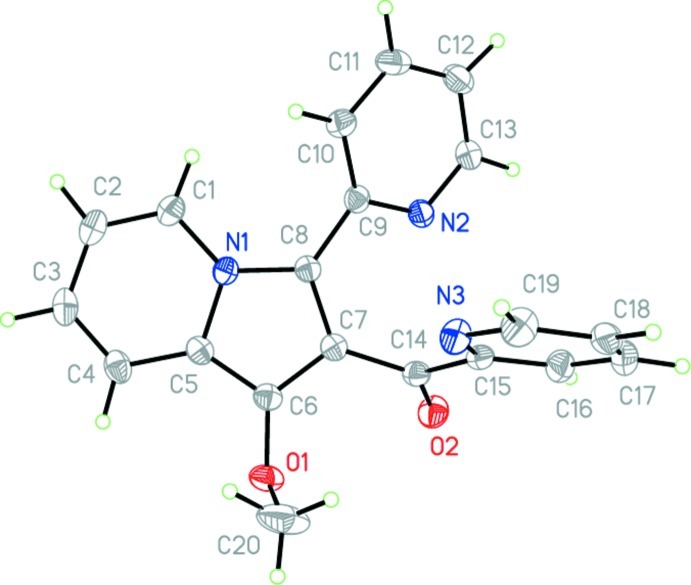
Molecular structure and numbering scheme of the title compound I; displacement ellipsoids are at the 40% probability level.

## supplementary crystallographic information

### Comment

Indolizines which offer manifold applications as *e.g.* dyes (Weidner *et al.*, 1989), pharmaceuticals (Singh & Mmatli, 2011), and spectral sensitizers (Gilchrist, 2001; Katritzky *et al.*, 1999; Vemula *et al.*, 2011; Sarkunam & Nallu, 2005; Weeler, 1985*a*,*b*) are rather scarce in nature whereas the reduced form of these heteroaromatic bicyclic compounds, the indolizidines, are quite common (Michael, 2007, and references therein). In these cases, *i.e.* the application of indolizines themselves or as intermediates in the synthesis of indolizidines, a well defined substitution pattern is required (Sarkunam & Nallu, 2005; Swinbourne *et al.*, 1978; Uchida & Matsumoto, 1976) and different transition-metal mediated and metal-free strategies for the synthesis of substituted indolizines have been investigated (Jacobs *et al.*, 2011; Kel'in *et al.*, 2001; Kim *et al.*, 2010; Liu *et al.*, 2007; Morra *et al.*, 2006; Seregin & Gevorgyan, 2006, Yan & Liu, 2007). The reaction of pyridinium *N*-methylides with acetylenes or with ethylenes in the presence of an oxidant causes limitations on the choice of substituents (Miki *et al.*, 1984; Padwa *et al.*, 1993) and applied oxidizers (Wei *et al.*, 1993). Another pathway, namely the cyclization reaction of 1,1-diacetyl-2-(2-pyridyl)ethylene in acetic acid anhydride at 60 °C or in refluxing dimethylsulfoxide, has also yielded substituted indolizines (Pohjala, 1974 and 1977).

### Experimental

[1-Hydroxy-3-(pyridin-2-yl)indolizin-2-yl](pyridin-2-yl)methanone (4.1 g, 13 mmol) was suspended in 120 ml of toluene. Potassium *tert*-butanolate (1.61 g, 14.33 mmol) was added and stirred for 18 h. To the resulting green reaction mixture, methyl iodide (890 µ*L*, 14.33 mmol) was added drop-wise. Then the solution was stirred for 24 h. The reaction mixture was filtered. The removal of all volatiles from the filtrate gave a dark yellow solid. Yield: 4.05 g of 1 (12.27 mmol, 94%). – ^1^H NMR (200 MHz, 303 K, [D_8_]DMSO): *δ =* 8.81 (d, 1H); 8.47 (d, 1H); 8.32 (dt, 1H); 7.91 (m, 2H); 7.55(dd, 2H); 7.45 (m, 1H); 7.18 (d, 1H); 7.11 (dt, 1H); 6.80 (dt, 1H); 6.67 (dt, 1H); 3.75 (s, 3H). – MS (EI): *m/z* (%) = 329 (92) [*M*]^+^; 314 (100); 298 (6); 286 (6); 143 (16); 106 (33); 78 (72). – Elemental analysis for C_20_H_15_N_3_O_2_ (329.36): calcd. C72.94, H 4.59, N 12.76 found C 72.25, H 4.67 N 12.57.

### Refinement

The hydrogen atoms of the methyl-group C20 were set to idealized positions and were refined with 1.5 times the isotropic displacement parameter of the carbon atom. The methyl groups were allowed to rotate but not to tip. All other hydrogen atoms were located by difference Fourier synthesis and freely refined.

### Crystal data

**Table:**

C_20_H_15_N_3_O_2_	*F*(000) = 1376
*M**_r_* = 329.35	*D*_x_ = 1.364 Mg m^−^^3^
Monoclinic, *C*2/*c*	Mo *K*α radiation, λ = 0.71073 Å
Hall symbol: -C 2yc	Cell parameters from 10598 reflections
*a* = 25.822 (2) Å	θ = 1.7–27.5°
*b* = 11.4406 (9) Å	µ = 0.09 mm^−^^1^
*c* = 11.3602 (7) Å	*T* = 183 K
β = 107.070 (4)°	Prism, brown
*V* = 3208.2 (4) Å^3^	0.05 × 0.05 × 0.05 mm
*Z* = 8	

### Data collection

**Table:**

Nonius KappaCCD diffractometer	2207 reflections with *I* > 2σ(*I*)
Radiation source: fine-focus sealed tube	*R*_int_ = 0.070
Graphite monochromator	θ_max_ = 27.5°, θ_min_ = 1.7°
phi– + ω–scan	*h* = −29→33
10598 measured reflections	*k* = −14→14
3667 independent reflections	*l* = −14→13

### Refinement

**Table:**

Refinement on *F*^2^	Primary atom site location: structure-invariant direct methods
Least-squares matrix: full	Secondary atom site location: difference Fourier map
*R*[*F*^2^ > 2σ(*F*^2^)] = 0.054	Hydrogen site location: inferred from neighbouring sites
*wR*(*F*^2^) = 0.132	H atoms treated by a mixture of independent and constrained refinement
*S* = 1.03	*w* = 1/[σ^2^(*F*_o_^2^) + (0.0651*P*)^2^] where *P* = (*F*_o_^2^ + 2*F*_c_^2^)/3
3667 reflections	(Δ/σ)_max_ < 0.001
275 parameters	Δρ_max_ = 0.22 e Å^−^^3^
0 restraints	Δρ_min_ = −0.28 e Å^−^^3^

### Special details

**Table:**

Geometry. All e.s.d.'s (except the e.s.d. in the dihedral angle between two l.s. planes) are estimated using the full covariance matrix. The cell e.s.d.'s are taken into account individually in the estimation of e.s.d.'s in distances, angles and torsion angles; correlations between e.s.d.'s in cell parameters are only used when they are defined by crystal symmetry. An approximate (isotropic) treatment of cell e.s.d.'s is used for estimating e.s.d.'s involving l.s. planes.
Refinement. Refinement of *F*^2^ against ALL reflections. The weighted *R*-factor *wR* and goodness of fit *S* are based on *F*^2^, conventional *R*-factors *R* are based on *F*, with *F* set to zero for negative *F*^2^. The threshold expression of *F*^2^ > σ(*F*^2^) is used only for calculating *R*-factors(gt) *etc*. and is not relevant to the choice of reflections for refinement. *R*-factors based on *F*^2^ are statistically about twice as large as those based on *F*, and *R*- factors based on ALL data will be even larger.

### Fractional atomic coordinates and isotropic or equivalent isotropic displacement parameters (Å^2^)

**Table:**

	*x*	*y*	*z*	*U*_iso_*/*U*_eq_	
O1	0.04698 (6)	0.09288 (12)	0.06993 (13)	0.0364 (4)	
O2	0.12184 (6)	0.12783 (12)	0.35467 (12)	0.0383 (4)	
N1	0.15233 (6)	0.26387 (13)	0.01130 (13)	0.0259 (4)	
N2	0.22178 (7)	0.32179 (13)	0.33083 (14)	0.0285 (4)	
N3	0.08352 (7)	0.41960 (14)	0.28790 (15)	0.0348 (4)	
C1	0.18167 (9)	0.29453 (18)	−0.06859 (18)	0.0315 (5)	
H1	0.2132 (10)	0.3465 (18)	−0.0350 (19)	0.042 (6)*	
C2	0.16708 (9)	0.25249 (19)	−0.18445 (19)	0.0365 (5)	
H2	0.1893 (9)	0.2805 (18)	−0.238 (2)	0.042 (6)*	
C3	0.12207 (10)	0.17639 (19)	−0.22799 (19)	0.0381 (5)	
H3	0.1124 (9)	0.1416 (18)	−0.313 (2)	0.039 (6)*	
C4	0.09303 (9)	0.14572 (17)	−0.15113 (17)	0.0328 (5)	
H4	0.0638 (9)	0.0923 (18)	−0.1749 (18)	0.032 (6)*	
C5	0.10777 (8)	0.18737 (16)	−0.02876 (17)	0.0272 (4)	
C6	0.08989 (8)	0.16467 (15)	0.07313 (17)	0.0276 (4)	
C7	0.12235 (8)	0.22698 (15)	0.17367 (16)	0.0254 (4)	
C8	0.16079 (8)	0.28941 (15)	0.13492 (16)	0.0248 (4)	
C9	0.20472 (8)	0.35985 (16)	0.21281 (16)	0.0265 (4)	
C10	0.22672 (9)	0.45894 (18)	0.17435 (19)	0.0323 (5)	
H10	0.2123 (9)	0.4926 (19)	0.095 (2)	0.041 (6)*	
C11	0.26848 (9)	0.51716 (19)	0.2576 (2)	0.0388 (5)	
H11	0.2828 (9)	0.5875 (19)	0.2335 (19)	0.045 (6)*	
C12	0.28677 (9)	0.47791 (19)	0.3779 (2)	0.0383 (5)	
H12	0.3157 (10)	0.513 (2)	0.442 (2)	0.060 (7)*	
C13	0.26168 (8)	0.38150 (18)	0.40973 (18)	0.0328 (5)	
H13	0.2725 (8)	0.3486 (16)	0.4960 (19)	0.033 (5)*	
C14	0.11818 (8)	0.22114 (17)	0.30094 (17)	0.0270 (4)	
C15	0.10492 (8)	0.32971 (16)	0.36106 (16)	0.0271 (4)	
C16	0.11300 (9)	0.3300 (2)	0.48702 (19)	0.0372 (5)	
H16	0.1298 (9)	0.2654 (18)	0.5342 (18)	0.031 (5)*	
C17	0.09646 (10)	0.4251 (2)	0.5409 (2)	0.0424 (6)	
H17	0.1002 (10)	0.425 (2)	0.629 (2)	0.053 (7)*	
C18	0.07292 (9)	0.5171 (2)	0.4673 (2)	0.0404 (6)	
H18	0.0603 (9)	0.5834 (19)	0.500 (2)	0.045 (6)*	
C19	0.06806 (10)	0.5113 (2)	0.3431 (2)	0.0423 (6)	
H19	0.0544 (10)	0.576 (2)	0.290 (2)	0.056 (7)*	
C20	−0.00098 (11)	0.1550 (2)	0.0648 (3)	0.0683 (8)	
H20C	−0.0300	0.0995	0.0643	0.102*	
H20B	−0.0117	0.2023	−0.0104	0.102*	
H20A	0.0055	0.2061	0.1369	0.102*	

### Atomic displacement parameters (Å^2^)

**Table:**

	*U*^11^	*U*^22^	*U*^33^	*U*^12^	*U*^13^	*U*^23^
O1	0.0330 (9)	0.0298 (8)	0.0470 (9)	−0.0081 (6)	0.0125 (7)	−0.0046 (6)
O2	0.0521 (10)	0.0285 (8)	0.0367 (8)	−0.0005 (7)	0.0169 (7)	0.0041 (6)
N1	0.0272 (9)	0.0271 (9)	0.0239 (8)	0.0024 (7)	0.0082 (7)	0.0018 (7)
N2	0.0298 (10)	0.0293 (8)	0.0260 (8)	−0.0013 (7)	0.0074 (7)	−0.0022 (7)
N3	0.0443 (11)	0.0292 (9)	0.0332 (9)	0.0047 (8)	0.0151 (8)	0.0022 (8)
C1	0.0309 (12)	0.0356 (12)	0.0302 (11)	0.0044 (10)	0.0123 (9)	0.0043 (9)
C2	0.0423 (13)	0.0419 (13)	0.0286 (11)	0.0085 (10)	0.0154 (10)	0.0058 (10)
C3	0.0507 (15)	0.0377 (12)	0.0248 (11)	0.0066 (11)	0.0091 (10)	−0.0013 (9)
C4	0.0414 (14)	0.0261 (11)	0.0272 (11)	0.0018 (10)	0.0042 (9)	−0.0014 (9)
C5	0.0292 (11)	0.0221 (9)	0.0290 (10)	0.0023 (8)	0.0067 (8)	−0.0016 (8)
C6	0.0280 (11)	0.0221 (10)	0.0326 (10)	0.0002 (8)	0.0087 (8)	−0.0015 (8)
C7	0.0273 (11)	0.0215 (9)	0.0272 (10)	0.0026 (8)	0.0079 (8)	0.0003 (8)
C8	0.0279 (11)	0.0203 (9)	0.0255 (9)	0.0010 (8)	0.0068 (8)	−0.0004 (8)
C9	0.0289 (11)	0.0256 (10)	0.0266 (10)	0.0021 (8)	0.0105 (8)	−0.0015 (8)
C10	0.0352 (12)	0.0305 (11)	0.0325 (11)	−0.0017 (9)	0.0119 (9)	0.0029 (9)
C11	0.0369 (13)	0.0300 (11)	0.0514 (14)	−0.0090 (10)	0.0159 (10)	−0.0018 (10)
C12	0.0356 (13)	0.0374 (12)	0.0399 (12)	−0.0067 (10)	0.0080 (10)	−0.0084 (10)
C13	0.0347 (13)	0.0337 (11)	0.0289 (11)	−0.0004 (9)	0.0076 (9)	−0.0032 (9)
C14	0.0263 (11)	0.0260 (10)	0.0282 (10)	−0.0023 (8)	0.0070 (8)	0.0019 (8)
C15	0.0280 (11)	0.0258 (10)	0.0297 (10)	−0.0048 (8)	0.0119 (8)	−0.0003 (8)
C16	0.0452 (14)	0.0382 (12)	0.0295 (11)	−0.0028 (11)	0.0129 (10)	0.0017 (10)
C17	0.0554 (16)	0.0420 (13)	0.0342 (12)	−0.0089 (11)	0.0199 (11)	−0.0096 (11)
C18	0.0443 (14)	0.0343 (12)	0.0492 (14)	−0.0100 (11)	0.0241 (11)	−0.0168 (11)
C19	0.0497 (15)	0.0322 (12)	0.0485 (14)	0.0073 (11)	0.0202 (11)	0.0026 (11)
C20	0.0404 (16)	0.0635 (18)	0.109 (2)	−0.0123 (14)	0.0349 (15)	−0.0344 (16)

### Geometric parameters (Å, º)

**Table:**

O1—C6	1.371 (2)	C8—C9	1.459 (3)
O1—C20	1.414 (3)	C9—C10	1.395 (3)
O2—C14	1.220 (2)	C10—C11	1.379 (3)
N1—C1	1.387 (2)	C10—H10	0.95 (2)
N1—C8	1.387 (2)	C11—C12	1.383 (3)
N1—C5	1.411 (2)	C11—H11	0.96 (2)
N2—C13	1.338 (2)	C12—C13	1.380 (3)
N2—C9	1.354 (2)	C12—H12	0.97 (2)
N3—C15	1.336 (2)	C13—H13	1.01 (2)
N3—C19	1.341 (3)	C14—C15	1.505 (3)
C1—C2	1.347 (3)	C15—C16	1.384 (3)
C1—H1	0.99 (2)	C16—C17	1.376 (3)
C2—C3	1.420 (3)	C16—H16	0.94 (2)
C2—H2	1.00 (2)	C17—C18	1.371 (3)
C3—C4	1.353 (3)	C17—H17	0.98 (2)
C3—H3	1.01 (2)	C18—C19	1.382 (3)
C4—C5	1.412 (3)	C18—H18	0.94 (2)
C4—H4	0.95 (2)	C19—H19	0.96 (3)
C5—C6	1.391 (3)	C20—H20C	0.9800
C6—C7	1.398 (3)	C20—H20B	0.9800
C7—C8	1.395 (3)	C20—H20A	0.9800
C7—C14	1.483 (3)		
			
C6—O1—C20	113.04 (16)	C9—C10—H10	122.9 (13)
C1—N1—C8	130.92 (17)	C10—C11—C12	119.5 (2)
C1—N1—C5	119.75 (16)	C10—C11—H11	119.9 (13)
C8—N1—C5	109.17 (15)	C12—C11—H11	120.4 (13)
C13—N2—C9	117.51 (17)	C13—C12—C11	117.9 (2)
C15—N3—C19	115.91 (17)	C13—C12—H12	116.9 (14)
C2—C1—N1	119.9 (2)	C11—C12—H12	125.2 (14)
C2—C1—H1	123.6 (12)	N2—C13—C12	124.12 (19)
N1—C1—H1	116.4 (12)	N2—C13—H13	113.5 (11)
C1—C2—C3	121.7 (2)	C12—C13—H13	122.3 (11)
C1—C2—H2	115.6 (12)	O2—C14—C7	120.69 (17)
C3—C2—H2	122.7 (12)	O2—C14—C15	119.34 (17)
C4—C3—C2	119.1 (2)	C7—C14—C15	119.79 (16)
C4—C3—H3	119.4 (12)	N3—C15—C16	123.41 (18)
C2—C3—H3	121.5 (12)	N3—C15—C14	117.44 (16)
C3—C4—C5	120.5 (2)	C16—C15—C14	119.08 (18)
C3—C4—H4	122.0 (12)	C17—C16—C15	119.3 (2)
C5—C4—H4	117.4 (12)	C17—C16—H16	121.3 (12)
C6—C5—N1	106.62 (15)	C15—C16—H16	119.4 (12)
C6—C5—C4	134.18 (19)	C18—C17—C16	118.4 (2)
N1—C5—C4	119.05 (18)	C18—C17—H17	120.9 (14)
O1—C6—C5	123.57 (16)	C16—C17—H17	120.6 (14)
O1—C6—C7	127.84 (17)	C17—C18—C19	118.5 (2)
C5—C6—C7	108.59 (17)	C17—C18—H18	121.3 (13)
C8—C7—C6	108.36 (16)	C19—C18—H18	120.2 (13)
C8—C7—C14	126.37 (16)	N3—C19—C18	124.4 (2)
C6—C7—C14	125.17 (17)	N3—C19—H19	115.0 (14)
N1—C8—C7	107.24 (15)	C18—C19—H19	120.6 (14)
N1—C8—C9	126.40 (17)	O1—C20—H20C	109.5
C7—C8—C9	126.14 (16)	O1—C20—H20B	109.5
N2—C9—C10	121.83 (17)	H20C—C20—H20B	109.5
N2—C9—C8	113.07 (16)	O1—C20—H20A	109.5
C10—C9—C8	125.07 (17)	H20C—C20—H20A	109.5
C11—C10—C9	119.08 (19)	H20B—C20—H20A	109.5
C11—C10—H10	117.7 (13)		
